# Measurement of AC loss down to 25 K in a REBCO racetrack coil for electrical aircraft motor

**DOI:** 10.1038/s41598-022-20625-6

**Published:** 2022-09-30

**Authors:** J. Kováč, Ľ. Kopera, E. Pardo, T. Melišek, R. Ries, E. Berberich, S. Wolfstädler, T. Reis

**Affiliations:** 1 Institute of Electrical Engineering, Slovak Academy of Sciences, V.V.I., Bratislava, Slovakia; 2 Oswald Elektromotoren GmbH, Miltenberg, Germany

**Keywords:** Electrical and electronic engineering, Applied physics

## Abstract

The development of full superconducting motors for electric distributed aircraft propulsion requires to test the stator coils at the operation temperature, usually between 20 and 40 K. Here, we study the AC loss of a test racetrack coil made of REBCO tape. We developed a measurement system within a non-metallic cryostat where a cryocooler cools the test coil in combination with liquid or solid nitrogen. We present transport AC loss measurements by electrical means down to 25 K for current amplitudes up to 140 A and frequency 18–576 Hz. The AC loss increased with second power with current, and did not depend on frequency or temperature. Later, we measured the AC parallel magnetization loss in a stack of tapes made of the same material as the coil, and in a stack of tapes without superconducting layer. The results in both samples is almost identical and presents the same behavior as the coil. We conclude that the main contribution to the AC loss in the tape stack and in the coil was from the magnetism of the Hastelloy substrate or buffer layers. Therefore, researchers need to take this into account in tape production and in superconducting motor design.

## Introduction

During the next decades, commercial air traffic is expected to grow, and hence aviation is an increasing source of greenhouse-effect emissions and pollution. Electric and hybrid-electric aircrafts with distributed propulsion can reduce CO_2_ and NO_x_ emissions by 75%, fuel burnt by 70% and noise by 71 db^1,2^. This technology needs motors with high specific power, having superconducting motors a high potential in this aspect. In addition, likely future increase of fuel prices will make fuel-saving technologies more attractive, if not mandatory. Superconducting motors are also promising for sea transport, thanks to the reduced weight, volume, and energy loss compared to their conventional counterparts. Superconducting generators share similar engineering challenges to motors and also present the same advantages. These generators are interesting for hybrid electric aircrafts and wind electric power generation, among other applications.

Superconducting machines (motors and generators) could be built with superconducting windings on the rotor, in the stator, or in both stator and rotor^1–9^. For superconducting stators, heat generation due to AC loss could be an issue in order to be able to keep the required low temperature for operation. Although REBCO coated conductor tapes (REBCO stands for *RE*Ba_2_Cu_3_O_7-x_, where *RE* is a rare-earth like Y, Gd, Sm or Eu) become superconducting below around 90 K, rotating machines need substantially lower temperatures to achieve the required high critical current density, *J*_*c*_; due to relatively high magnetic fields (around 1–3 T). The temperature range between 20 and 40 K is interesting thanks to a compromise between high *J*_*c*_ and sufficiently large temperatures to be cooled by liquid hydrogen or cryocoolers^1,2^. Liquid hydrogen cooling is especially interesting, since it could also serve as fuel^1,2,10^. Although superconducting MgB_2_ stators could also operate at liquid hydrogen temperature (20 K), REBCO offers higher engineering current densities. REBCO also enables higher cryogenic flexibility, since the critical current density is still high at significantly higher temperatures than 20 K, in contrast to MgB_2_ wires. If cooled by liquid hydrogen, the windings will be at temperatures above 20 K, since heat dissipation in the conductors will cause temperature gradients. In MgB_2_, heating due to AC loss is kept low by filamentation and twisting, while REBCO stators can achieve low AC loss thanks to magnetic shielding either due to iron teeth^3^ or superconductor stacking effects^2,11^.

The development of superconducting machines requires the construction of demonstrators and prototypes, after the superconductor winding technology is reliably well tested. Then, there is a need to characterize and test superconducting windings, typically racetrack coils, at temperatures down to 20 K. There is also a need to study in depth the AC loss behavior at variable temperature in simplified systems, such as racetrack coils, in order to be able to understand the main contributions to the AC loss.

In a full superconducting motor, the highest AC loss will occur at the stator. The base frequency highly depends on the motor architecture, but it will often be of the order of hundreds of Hz for an aircraft propulsion motor^2^. However, there could be higher harmonics in the current due to the switching frequency of the current power source. Then, coil testing should ideally be done to frequencies of up to few hundreds of Hz or few kHz.

Although there have been many works about AC loss measurement of high-temperature superconducting coils at liquid nitrogen temperatures (77 K) at power frequencies^7,12–17^ and high frequencies^18–20^, there exist very few works about AC loss studies in coils at the 20–40 K temperature range due to the involved cryogenic challenges. The pioneering work in Ref.^21^ uses a cryocooler to achieve temperatures down to 30 K. That set-up measures coils of few turns in low-inductive transformer configuration, although very little information is disclosed. Article^22^ also uses a cryocooler to measure the magnetization AC loss in wires and tapes down to 15.5 K and frequencies from 2.3 up to 1152 Hz. Reference^23^ presents an interesting system to measure AC loss down to 10 K and frequencies up to 18 kHz. However, only measurements on a single wire at frequencies up to 20 Hz are reported. Round REBCO coils at temperatures down to 20 K are measured in Ref.^24^ but the experiments are limited to low power loss, below 1.3 W. Other systems with helium gas cooling can extract higher heat power, as done in Ref.^25^ for around 100 W. That work measures a considerably large coil but at low frequencies, up to 2 Hz. Apart from these works at low temperatures, the measurements in Refs.^26,27^ at 77 K are highly remarkable. These experiments use a system that generates high magnetic inductions (more than 500 mT amplitude) at moderate frequencies, up to 200 Hz. Although these works mention that the system could also measure coils, they only report results for tapes and cables. In addition, Ref.^6^ measures the AC loss in a stator pancake coil at 77 K in the environment of an axial flux motor.

AC loss could be measured by two different techniques: calorimetric^6,15,21,26,27^ or electric^12–16,18,19,21–25^. Electric measurements are more accurate than calorimetric, enabling to determine the AC loss also at low AC excitations. In addition, they can be applied to any temperature, being ideal for variable temperature studies. However, they are sensitive to AC loss in induced currents in surrounding metals, which requires non-metallic cryostats that complicate the experimental set-up. Calorimetric measurements avoid these complications, since they are local. Their disadvantages are that they often require a boiling liquid, which limits the temperature (nitrogen at 77 K or helium at 4.2 K), and are less accurate than electric methods.

In this article, we present an experimental system to electrically measure the AC loss in racetrack or pancake coils at temperatures from 25 to 300 K, direct input currents up to 300 A, alternating current amplitudes up to 140 A and frequencies up to 576 Hz (see “Measurement set-up” section). We achieve low temperatures by a cryocooler via conduction cooling, improved by solid nitrogen. Then, the unique aspects of the system are the high temperature range (25 to 300 K), high frequency (up to 576 Hz, but might be increased to few kHz), high current amplitude (140 A, with a potential to reach 300 A), high sensitivity, relatively large sample size (up to 254 mm diameter), and the possibility to use innovative cryogenic media like solid nitrogen. With this system, we analyze and test a racetrack coil made of several REBCO tapes in parallel, obtaining interesting results (“Experimental results” section). Unexpectedly, the main AC loss contribution at the measured current amplitudes, which are well below the coil critical current, is consistent to residual ferromagnetic hysteresis loss from the metallic substrate or buffer layers (“Transport AC loss in coil at currents up to 140 A in amplitude” section). In order to support this conclusion, we made further measurements on stacks of tapes under sinusoidal applied magnetic fields (“Magnetization AC loss in stack of tapes” section). Although the main measurement results here were already presented at international conferences, such as Ref.^28^, they were not reported in any article.

## Measurement set-up

### Superconducting test coil

The test coil was made of SuperOx REBCO tape of 4 mm width and around 0.1 mm thickness, which was delivered in 2019. The racetrack coil, of 12 turns, used a conductor made of 2 tapes in parallel electrically insulated from each other. The racetrack coil is of 245 mm length and 65 mm width. The cross-section of one of the straight segments is 7.6 mm wide. The typical dependence of the critical current density, *J*_*c*_, with the magnetic induction, *B*, and its orientation to the tape normal for several temperatures (20 to 40 K) is in Ref.^2^.

### Cooling system and thermal behavior of the test coil

The expected operating temperature of the superconducting aircraft engine is around 25 K. This is 5 K above the boiling temperature of hydrogen in order to ensure efficient heat extraction from the coil.

**Figure 1:**
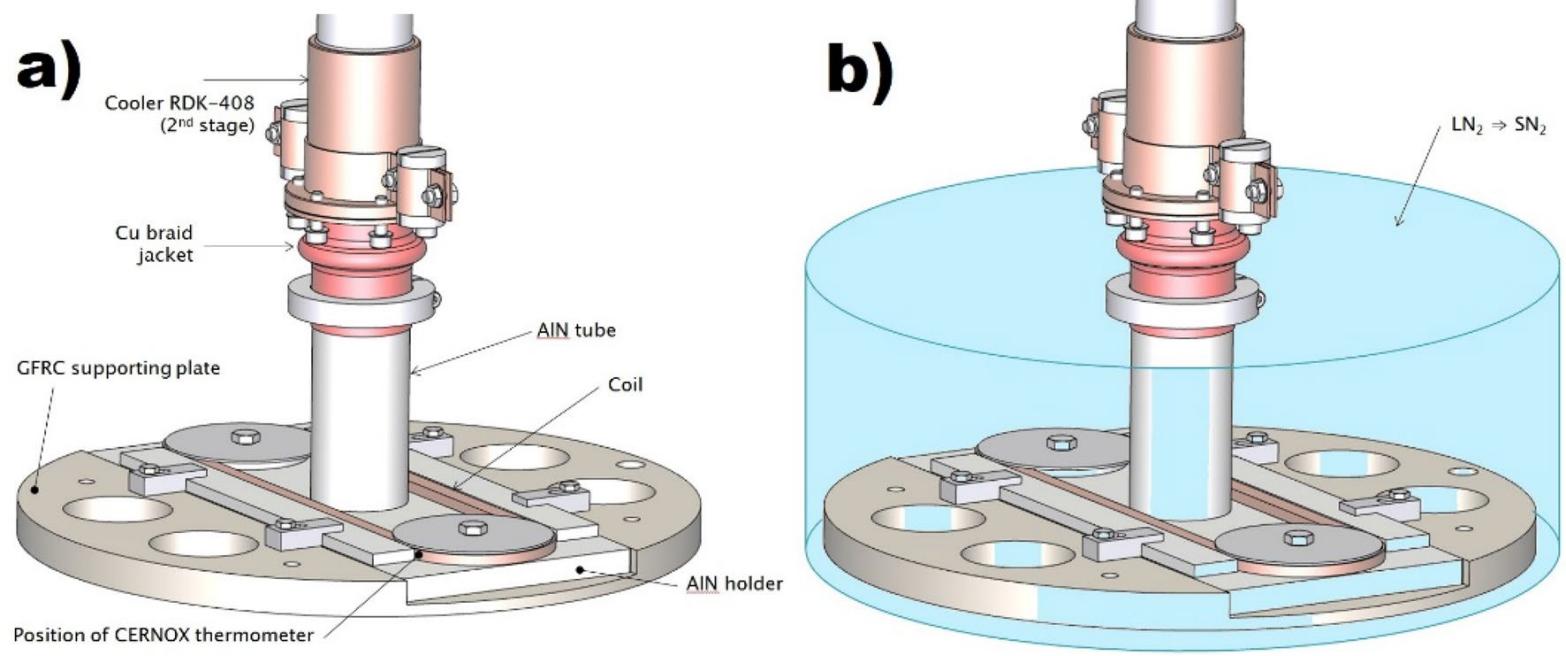
(**a**) Sketch of the coil assembly for AC loss measurements in vacuum in the measuring set-up prior inserting to the cyostat. (**b**) Approximate level of 6 L of solid nitrogen sub-cooled to 23 K.

Therefore, a goal of our experiments was to perform AC loss measurements at down to 25 K. The main restrictions of the cooling system design were: (a) the need of non-metallic materials in the proximity of the coil and in the cryostat; (b) the low cooling capacity of the available RDK-408 cryocooler. The AC loss measurements of the coil at temperatures below 40 K in vacuum are possible only in case of a fast and reliable transfer of the heat generated in the coil to the cooler. The cooler’s cold head is made of solid copper and, to minimize its influence to AC loss measurements, it should be located at a certain distance from the measured coil. The coil is fixed to Aluminum Nitride (AlN) ceramic holder supported with glass fiber reinforced composite (GFRC) plate, which is mounted to the cooler at a distance around 120 mm (Fig. 1). The heat transfer from the coil to cooler is provided through a thick-wall AlN tube of outer diameter 40 mm and length 120 mm; whose bottom and top sides are mounted to the AlN holder and a copper braid jacket, respectively (Fig. 1).

**Figure 2:**
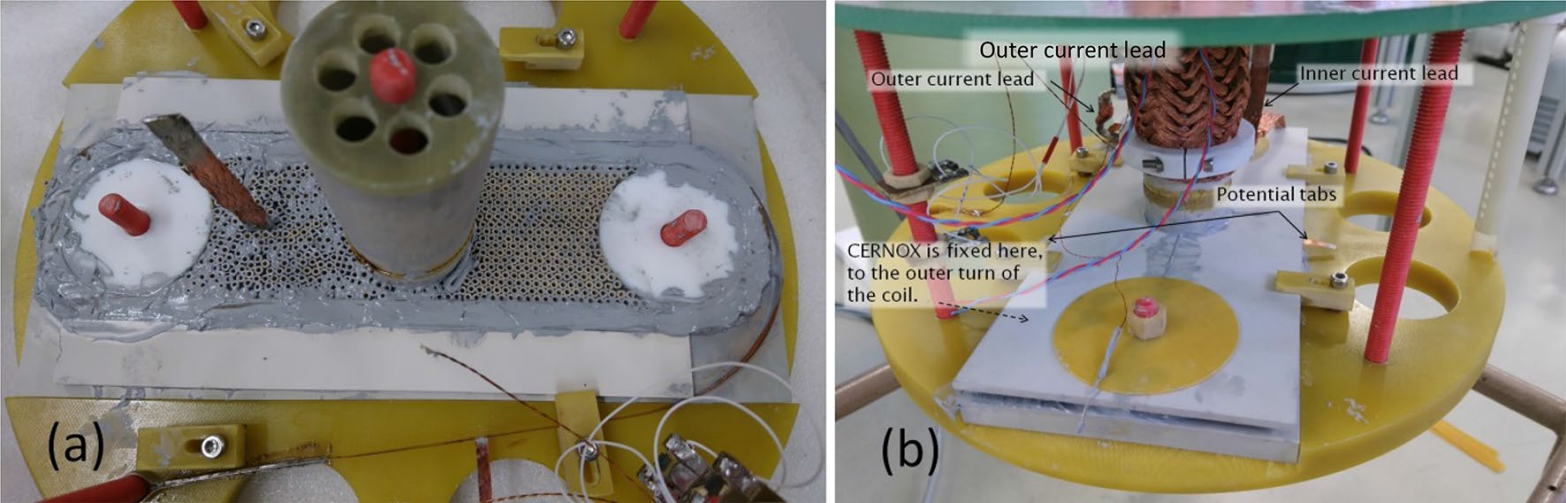
(**a**) The coil filled by ceramic beads and covered by thermal conductive grease. (**b**) The finished measuring setup prior inserting into the cryostat.

To improve the heat transfer from the coil to AlN holder, the coil was covered by around 1 mm thick layer of high thermal conductivity grease (Fig. 2a). Cernox thermometer was fixed directly to the surface of the top layer of the HTS tape winding at a distance around 20 cm from the current lead (Fig. 2b). To improve the heat transfer from the coil, the holder assembly was filled by 6 L of LN_2_ (liquid nitrogen) to a level around 10 cm above the coil surface. The LN_2_ was then sub-cooled to 64 K by decreasing the pressure in the cryostat chamber and further cooled down by the cryocooler, which solidified the nitrogen (Fig. 1b). The lowest temperature measured on the coil sample in solid nitrogen was 23.4 K.

Initial AC loss measurement in SN_2_ (solid nitrogen) showed relatively good heat transfer from the coil to the cooler. However, repeated measurements showed stepwise reduction of the heat transfer from the coil. The main problem is to preserve a good contact between the coil surface and the surrounding nitrogen ice. The surface of the solid nitrogen naturally sublimates and freezes on the 2nd stage of the cryocooler which is at lower temperature (at around 10 K) than the coil surrounded by the nitrogen ice. Any heat generated in the coil increases the sublimation ratio of the ice that is in direct contact with the measured sample and, consequently, forms a gap between the neighboring surfaces (dry-out effect). This is the most serious problem of reliable heat transfer, since there is no way to recover the initial good thermal contact—just to heat-up the experimental setup to LN_2_ temperature and cool it down again—but this process takes long time. In order to minimize the dry-out effect, we filled the free space between the coils by a single layer of ceramic tubular beads (Fig. 2a). Thus, LN_2_ penetrates in the beads holes and interstices and later solidifies, forming a composite of high thermal capacity and conductivity.

To improve the heat transfer also from the top of coil, 3 mm thick AlN plate was added to the top surface of the coil (Fig. 2b).

**Figure 3:**
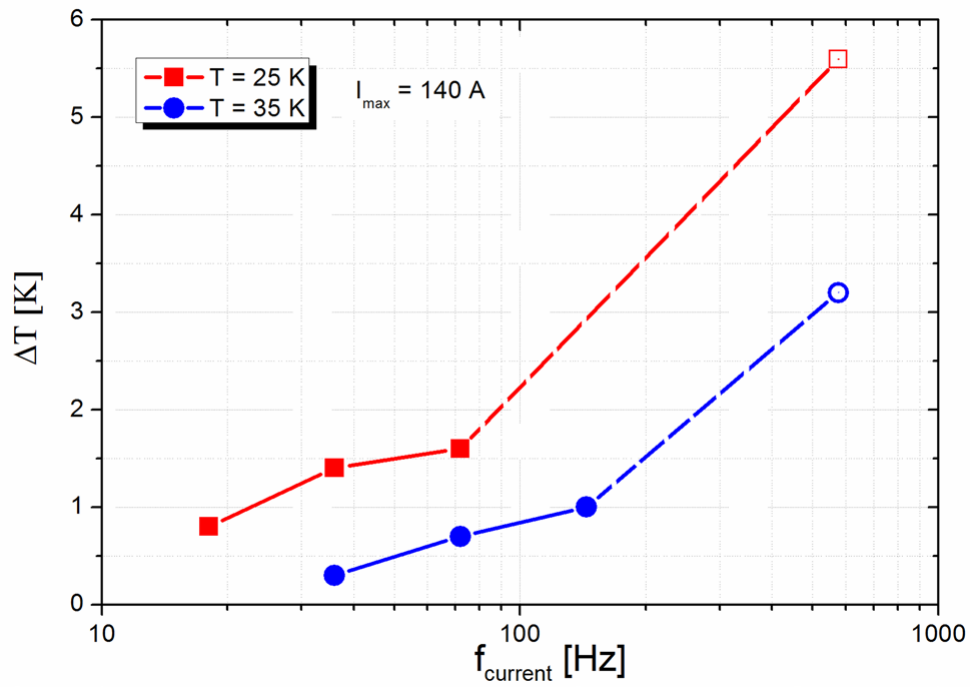
Thermal behaviors of test coil at various current frequencies for current amplitude of 140 A (100 A RMS). For the highest frequency 576 Hz (open symbols), the maximum achievable current amplitude was 127 A (90 A RMS) because of thermal stability issues.

Figure 3 describes the thermal behavior of the test coil. The temperature increase Δ*T* is the maximum value measured after 1 min of inserting transport current of amplitude *I*_max_. For AC currents, Δ*T* increases with the current amplitude. There was also clear dependence on frequency at constant current amplitude. In DC regime there was not significant temperature rise even at higher currents. The maximum raise of temperature during DC measurement up to 290 A was approximately 0.16 K (at temperature 23.6 K). We did not see any increase of the coil temperature up to 200 A, and only negligible temperature increasing was measured at 250 A and 290 A (Δ*T* = 0.10 K and 0.16 K, respectively). Considering different thermal behavior in DC and AC regime respectively, we can conclude, that the heating is caused by AC loss.

### Transport AC loss measurement system

AC loss measurements via Lock-In technique^29,30^ current sensing requires very precise phase detection. In our set-up, we used a precise, high current shunt resistor. In this case, the inductive part of the shunt impedance cannot be neglected. Any phase error due to wrong phase detection during current measurement can significantly disturb the results of AC loss measurements. Therefore, precise current phase detection has crucial impact for the measurement reliability. We have made several efforts to measure the current by shunt resistor without any phase shift (see the Supplementary Material).

The total inductance of the coil was measured and calculated by means of COMSOL software. The computed inductance of (190 µH) agrees relatively well with the measured one (200 µH). This agreement confirms that there are no shortcuts between turns in the winding. At high frequencies or amplitudes of transport current, induced voltages due to the coil inductance can reach voltage amplitudes which are far above the high limit of the Lock-in amplifier input. Therefore, we used a voltage divider to reduce the high voltage to a suitable low level (see the Supplementary Material).

We power the coil by AC transport current using a wave generator augmented by a 5 kW audio amplifier QSC5050. The amplifier is intended to be used with 2 Ω load impedance and, therefore, we cannot connect the test coil directly to that equipment. Instead, it is necessary to accommodate the load impedance of the measuring setup by a current transformer and capacitive compensation. To reach the required current amplitudes at various frequencies, the transformer ratio and the compensation capacitance have to be set separately for each frequency. As a wave generator, we use the internal signal generator of a Lock-in amplifier Signal Recovery 7625.

## Experimental results

### DC measurements of coil

To prevent the coil damage by destructive quench (this case is even more critical in the case of parallel tapes in winding), before AC loss measurements at current amplitudes higher than 200 A, DC test measurements were done to find out the thermal stability of the coil. We measured the coil with several current steps of DC transport current (*I*_max_ = 100 A, 150 A, 200 A, 250 A and 285 A) at temperature 23.6 K. Each current transfer ramp took around 1–2 min. Standard four probe method of I-V measurement was used. As a power supply, we used a Sorensen DCS 8–350 source. Voltage on the coil taps was measured by a Keithley 2182A nanovoltmeter. The maximum rise of temperature during DC measurement was approximately 0.16 K (at temperature 23.6 K). The results show that the coil is able to carry DC transport current up to 285 A without any visible electric or thermal instability. We did not see any increase of the coil temperature up to 200 A, and only negligible temperature increase was measured at 250 K and 285 K (Δ*T* = 0.10 K and 0.16 K, respectively).

### Transport AC loss in coil at currents up to 140 A in amplitude

**Figure 4:**
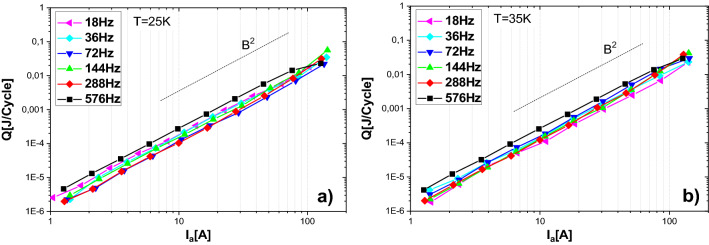
Coil AC loss per unit cycle versus transport current amplitude at temperatures 25 K and 35 K.

Measured transport AC loss per unit cycle versus transport current amplitude at frequencies 18, 36, 72, 144, 288 and 576 Hz are shown in Fig. 4a,b for temperatures of 25 and 35 K, respectively. The loss is roughly proportional to *B*^2^ in the whole current range, at both temperatures as well as at each measured frequency (the explanation of the causes of this behavior is explained close to the end of this section). The presented results indicate, that in agreement with expectation (as well as measurements in DC regime), current maximum of 140 A in amplitude is much lower than the expected critical current of the coil. Indeed, DC measurements indicate that the critical current is above 285 A (see “DC measurements of coil” section). There are some variances of slope near to maximum current amplitudes. We expect two possible causes of this issue. The first could be temperature instabilities in the coil. Indeed, both AC loss from the coil and heating from the current leads increase with current amplitude. When the heat generation approaches to the cooling power of the cryocooler, the temperature becomes unstable and non-uniform (the temperature is regulated from the signal of a single temperature sensor in the coil). The other reason could be errors in the phase settings. Although the phase is accurately set at the beginning of the measurements for each amplitude and frequency, at high amplitudes the temperature of the whole current leads (both inside and outside the cryostat) increases during the measurement, which increases the resistance of the LR circuit formed by the coil and current leads. Transformer core heating might also influence the phase.

**Figure 5:**
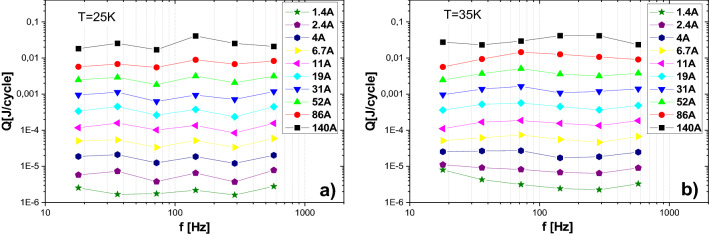
Frequency dependence of transport AC loss per unit cycle of the coil at temperatures 25 K and 35 K.

Frequency dependences of transport AC loss per unit cycle at various current amplitudes ranged from 1.4 A up to 140 A are shown in Fig. 5a,b for temperatures of 25 and 35 K, respectively. At both temperatures, as well as at the given current amplitudes, there is no significant frequency dependence of AC loss per cycle (hence, the power loss in W roughly increases linearly with frequency). Such behavior indicates that the main contribution to the AC loss does not come from the eddy current loss in metal parts of the coil. In the same way, influence of coupling current loss (despite the parallel winding) are not visible in the given frequency range.

**Figure 6:**
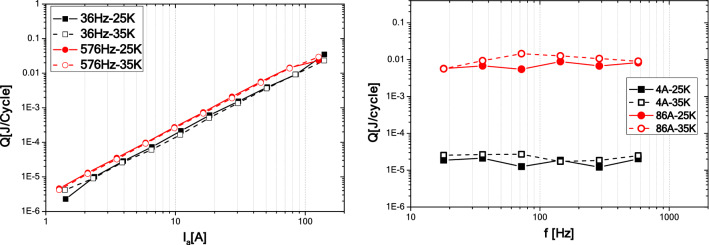
Comparison of coil AC loss dependence versus transport current and frequency, measured at temperatures 25 K and 35 K.

Finally, comparison of AC loss dependence versus transport current and frequency, at temperatures 25 K and 35 K are shown in Fig. 6. This comparison confirms that there are only negligible differences between the measurements at both temperatures.

From all the AC loss measurements on the coil, we conclude that: (a) there is weak or negligible frequency dependence; (b) there is weak or negligible difference between results at temperature 25 K and 35 K respectively; (c) the slope of AC loss versus current in logarithmic scale is approximately two at both temperatures, whole frequency range, and whole range of transport currents. Such results are in several points in opposite to theoretical assumptions, as explained below. The slope in second order is symptomatic for eddy current loss in metallic parts of superconducting composite (copper, silver and Hastelloy®)^31^. In the case of multifilamentary superconductor or multiple single core superconductor or tapes that are electrically connected (coupled), the coupling current loss could also play a role. Again, the slope of the AC loss should be 2. For both mentioned type of loss significant frequency dependence is expected. However, our results are comparably the same in the whole frequency range, and hence they are frequency independent. This frequency independent loss behavior indicates dominance of superconducting hysteresis loss. In the other hand, a slope between 3 and 4 is expected for racetrack coils^31–33^. In principle, the power 2 dependence could be explained by inhomogeneous critical current density of the superconducting layer. If the current density near to edges of the tape is lower than in the center, the slope of the hysteresis loss decreases, which could cause a slope close to 2^34^. The reason is that for low current amplitudes, there appears current density around *J*_*c*_ only close to the edges, where the critical current density could be lower than the average, causing a relative increase of the AC loss. For any type of homogeneity, the decrease of *J*_*C*_ by increasing the temperature from 25 to 35 K should cause a significant increase in superconducting hysteresis AC loss. However, measurements show practically the same AC loss. Therefore, neither eddy currents nor superconducting hysteresis can be the cause of the measured AC loss.

Another reason of observed behavior could be caused by magnetic hysteresis in the metal substrate of the superconducting tape. For the magnetic hysteresis, slope 2 has also been experimentally observed^35–37^ and because the loss per cycle does not depend on the cycle duration, it is frequency independent. Moreover, if the magnetic properties do not change with temperature, the AC loss will not change with temperature as well. Considering this, our hypothesis to describe the obtained experimental results is that the magnetic hysteresis in the substrate is dominant. To confirm this explanation, we measured the magnetization AC loss in a stack of four insulated tapes (the same as used in the examined coil) by a different set-up (“Magnetization AC loss in stack of tapes” section). To show directly the impact of substrate layer to the overall loss, we made also identical four tapes stack without superconducting and copper stabilizing layers. Such modification excludes also the possibility of discrepancies by eddy current loss in the copper layer, where the electric conductivity is high. The results confirm that the measured AC loss of the test coil is strongly affected by the magnetic properties of the tape substrate.

### Magnetization AC loss in stack of tapes

Although for the superconducting parts the magnetization and transport AC loss configurations are significantly different, the qualitative behavior of the AC loss from the residually magnetic substrate is the same. The reason is that in the coil practically all the current flows on the superconducting and stabilization layers, being the substrate effectively current-free. Modelling by our own software^3,14^ shows that at the maximum AC current (140 A) the maximum magnetic field in the coil are around 200 and 170 mT in the directions perpendicular and parallel to the tapes, respectively. As our results will show below, the substrate AC loss practically does not depend on the direction of the applied magnetic field. As well, our magnetic AC loss measurements reach up to around 100 mT, which is of the same order of magnitude as the maximum magnetic field in the coil. Then, the AC magnetization measurements are expected to provide the same qualitative behavior of the substrate AC loss as in the AC transport measurements of the coil.

The magnetization AC loss of the stack of four insulated tapes were electrically measured by the calibration free method^38^ in a set-up that cools the sample by a cryocooler down to 20 K^22^. The sensitivity of this system is very high, which is able to obtain accurate measurements down to around 10^–9^ J/cycle/m in samples of around 100 mm length^39^. High sensitivity and accuracy are achieved by fine phase compensation by pick-up coils and removal of empty-sample signal. To obtain the same conditions as during the test coil measurements, all experiments were performed at temperatures of 25 K and 35 K. To show directly the impact of the substrate, identical four tapes stack of the same dimensions, insulation distance but without superconducting layer was measured under the same conditions. Measurements were performed in external AC field amplitudes ranged from 0.1 up to 100 mT with frequencies of 72 Hz and 144 Hz. The field orientation was parallel to the tapes plane. Moreover, to prove the impact of substrate layer and exclude the possibility of influence of superconducting layer residues, measurements were performed up to temperatures above the critical one. We removed the superconducting layer as follows. First, we chemically etched the copper stabilization layer by FeCl_3_. Later, we removed the superconducting layer by polishing with sandpaper. The total length of the tape stack as well as Substrate stack were 70 mm.

**Figure 7:**
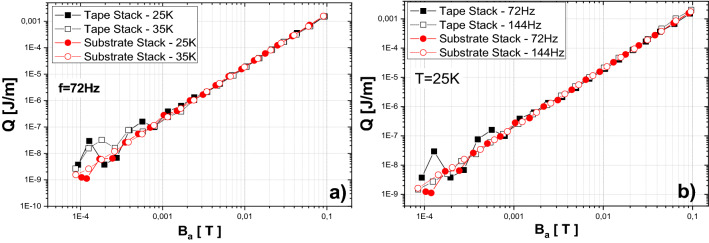
(**a**) Comparison of magnetization AC loss measurement in parallel field of insulated tape stack versus measurements of same stack without superconducting layer at 25 and 35 K (shown quantity is AC loss per cycle). (**b**) The same comparison at temperature 25 K and two frequencies 72 Hz and 144 Hz.

Figure 7a shows the measurements at two temperatures, 25 and 35 K. Measurements were performed at 72 Hz frequency and the AC field amplitude was ranged from 0.1 mT (10^–4^ T) up to 100 mT (10^–1^ T). As can be seen from the results, loss per cycle is increasing with second slope order at whole range of external field (except noise bellow 1 mT). Moreover, different temperatures lead to negligible differences in AC loss results. But much more relevant is the comparison between results of magnetization AC loss measured in stack of superconductive tapes, and the AC loss measured in the stack of tapes without superconducting and copper layers. As can be seen from Fig. 7a, magnetization AC loss of non-superconducting stack (named Substrate Stack) are practically identical in the whole range of field as that measured in stack of superconducting tapes at both 25 and 35 K. Again, the ripple below 10^–3^ T can be considered as measurement error due to very small measured signal. Figure 7b shows the comparison of both examined stacks at temperature 25 K and two frequencies of applied field, 72 and 144 Hz. As in the previous case, the results for the superconducting stack and the substrate stack are the same. In addition, the results do not differ with frequency.

**Figure 8:**
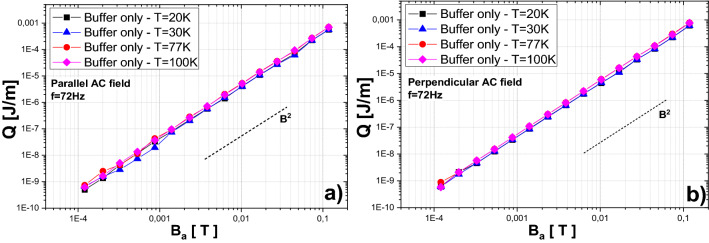
Comparison of measured magnetization AC loss per cycle under perpendicular and parallel field of tape stack without superconducting layer at temperatures up to 100 K.

To exclude the possible impact of superconducting layer residues, the magnetization AC loss measurements of stack in parallel as well as perpendicular field at temperatures up to 100 K (above critical one) were performed. The results are shown in Fig. 8a,b for parallel and perpendicular applied magnetic field, respectively. As can be seen, there is no significant difference between the results measured at 20 K and 100 K in both field orientations. Such behavior excludes the presence of superconducting layer in the substrate stack and confirms the hypothesis of magnetic hysteresis loss in substrate material.

The magnetization measurements agree with the hypothesis of magnetization hysteresis loss in metallic substrate layer. There are still visible also in the stack without superconducting layer, therefore the AC loss cannot have superconducting hysteresis origin. Moreover, critical current density necessarily depends on temperature, and hence superconducting hysteresis loss have to also depend on temperature and vanish above the critical temperature. On the other side, the magnetic properties of the metallic substrate do not necessarily change in the temperature range between 25 and 35 K, which can explain no difference in our results measured in both stacks. As was mentioned above, the value of hysteresis loss (magnetic as well as superconducting hysteresis) per cycle does not depend on the frequency. This frequency independent behavior is in agreement with the theoretical assumption for both cases; magnetic hysteresis as well as superconducting hysteresis loss. In other hand, third or fourth order slope is typical for superconducting hysteresis loss, while second slope order is admissible for loss of magnetic material. Considering experimental results of magnetization AC loss measurements of both tape stacks (superconducting tapes as well as substrate only), we can conclude, that the substrate has a crucial impact to the overall loss. The contribution of superconducting layer is negligible in parallel field, and hence the decisive impact to the overall loss of the tape stack is from the substrate material. We can also assume the same behavior for whole test coil. In addition, the measured AC loss is between one and two orders of magnitude higher than the parallel AC loss of a similar stack made from a different producer (SuperPower)^40^.

## Conclusion

For experimental study of AC loss in motor racetrack test coil, a unique measurements system for transport AC loss measurements was developed. For this purpose, we designed cryocooler cooled system with a non-metallic cryostat that allows to measure transport loss in liquid nitrogen, supercooled nitrogen at low pressure, as well as in solid nitrogen at a temperature lower than solidification. The measurements in test coil were performed at temperatures down to 25 K. Especial high attention was paid to the phase setting and its accurate measurements, which has crucial impact to the results reliability. A special non-inductive shunt resistor was used for current measurement and an active voltage divider for elimination of inductive part of measured signal. Transport AC loss measurements were performed in current range up to 140 A and frequency ranged from 18 up to 576 Hz. It was observed that the resulting transport AC loss of the test coil increased with second power with current. Moreover, there was lack of both frequency and temperature dependence. One possible explanation of such behavior is the impact of magnetic hysteresis loss in the substrate, or even the buffer layer. To confirm this hypothesis, magnetization AC loss measurement in stack of tapes used for coil production as well as in stack of tapes without superconducting and stabilizing layers were performed. Both stacks were measured at the same temperature as for the whole coil in external AC field oriented in parallel to the tape’s plane. The results show that AC loss in the tape stack increased with second power with current amplitude and, as in the case of whole coil, there was not significant impact of frequency and temperature. Moreover, measurements in stack of tapes without superconducting layer, in which the superconducting hysteresis loss is excluded, led to similar results. Considering this, we conclude that the crucial impact to the overall AC loss in both tape stack and test coil is the substrate material. Comparing to previous measurements of stacks from another producer (SuperPower), we see that the AC loss per unit length of the SuperPower tape is at least one order of magnitude lower^40^. Thus, this contribution from the substrate magnetism might be particular of SuperOx tapes, or even a particular batch of this producer. Nevertheless, our work evidences that the apparently low magnetism of the substrates of REBCO tapes from IBAD-like route should be taken in both tape manufacturing and device design into account. In motors for aviation, this is important when the stacking effect is exploited, where superconductor loss is significantly suppressed but not the contribution from the substrate. This also applies for cable configurations with striated tapes, such as CORC^41^. In addition, potentially magnetic buffer layers like MgO might play a role in the AC loss, which could deserve future study.

## Supplementary Information

Supplementary Information.

## Data Availability

The datasets generated during and/or analysed during the current study are available from the corresponding author on reasonable request.
