# Supplementary material for: Measurement of AC loss down to 25 K in a REBCO racetrack coil for electrical aircraft motor
Source: Sci Rep. 2022 Sep 30;12:16454. doi: 10.1038/s41598-022-20625-6 (PMC9525724; doi:10.1038/s41598-022-20625-6)
Supplement: Supplementary file 1 — Supplementary Information. [file 41598_2022_20625_MOESM1_ESM.pdf]

Supplementary material to:
Measurement of AC loss down to 25 K in a REBCO racetrack coil for electrical aircraft motor

J. Kováč[1], Ľ. Kopera[1], E. Pardo[1*], T. Melišek[1], R. Ries[1], E. Berberich[2], S. Wolfstädler[2], T. Reis[2]

1. Institute of Electrical Engineering, Slovak Academy of Sciences, v.v.i., Bratislava
2. Oswald Elektromotoren GmbH, Germany

* corresponding author: enric.pardo@savba.sk

A. Phase correction in AC loss measurements

This supplementary material provides details on the phase correction of transport AC loss experiments of superconducting coils by electric means. Accurate phase correction is key for the correctness and reliability of the measurements.

A.1 Calibration of shunt's phase shift

The basic principle of eliminating the self-induction of the shunt resistor is based on its compensation by another inductance. Therefore, we added a compensation "coil" (one turn shaped as a figure eight) in order to minimize the inductive part of the shunt's impedance (in the best case to zero) by precisely changing its shape and position. We find the optimum shape of the compensation coil by comparing to a precise non-inductive shunt (Tinsley London type 1682) that serves as a reference. Unfortunately, the current in this non-inductive reference resistor is limited to 50 A, and hence it cannot be directly used for high current measurements. Then, the reference shunt and the calibrated resistor were connected in series to the power supply to carry the same current. Four probe method and Lock-in amplifier were used for signal measurement. The first channel of Lock-in amplifier measured voltage signal from the non-inductive component. The phase shift of the first channel was set to zero. Then, on the second channel, the phase shift of the calibrated high current shunt resistor was zeroed by adjusting the area and position of the compensation coil. This calibration procedure was made separately for several ranges of the current amplitude. Similarly, the phase shift had to be set for each measured frequency.

A.2 Using the voltage divider.

As mentioned earlier, the inductive voltage part of measured signal due to coil inductance can reach high values, which overcome the input range of the lock-in amplifier. We reduce the voltage without introducing additional phase shift by an active, galvanic insulated, optical voltage divider Cal Test Electronics CT4072. This unit is intended to work up to very high frequencies (of the order of 10^8 Hz) and for low frequencies below 10^3 Hz it works with a negligible low phase shift. Measurement accuracy was verified by test measurements at frequencies and current values usable with, as well as without, the voltage divider (Figure 1). Since both measurements agree, we conclude that the voltage divider does not introduce phase shift.

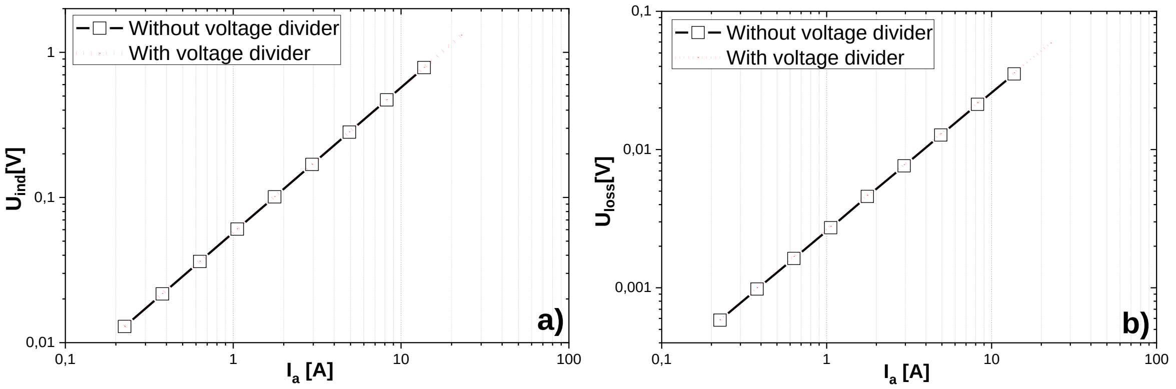

Figure 1: The voltage divider does not introduce errors in both inductive and loss voltages (U_{ind} and U_{loss}) of the coil, since the signals with and without divider are the same.

A.3 Additional Phase error corrections

Standard Lock-in AC loss measuring technique is based on separation of inductive part and loss part of the measured signal [1,2]. The part in phase with transport current (minus sign) represents the inductive voltage, and the quadrature component represents the loss part of the measured signal. In the case of coils, the inductive part is much higher than the loss one. For this reason, as was written above, we have paid a lot of attention to eliminate the phase shift during both current and signal measurements. For the purpose of supplementary measurement error correction, we have log also the phase error during measurements, which was consequently used for corrections. The phase error was obtained as an absolute value of the phase of measured current after setting the internal Lock-in coordinates system into phase with current (which is zero in ideal case). The example of results without and with calculated corrections are shown in Figure 2.

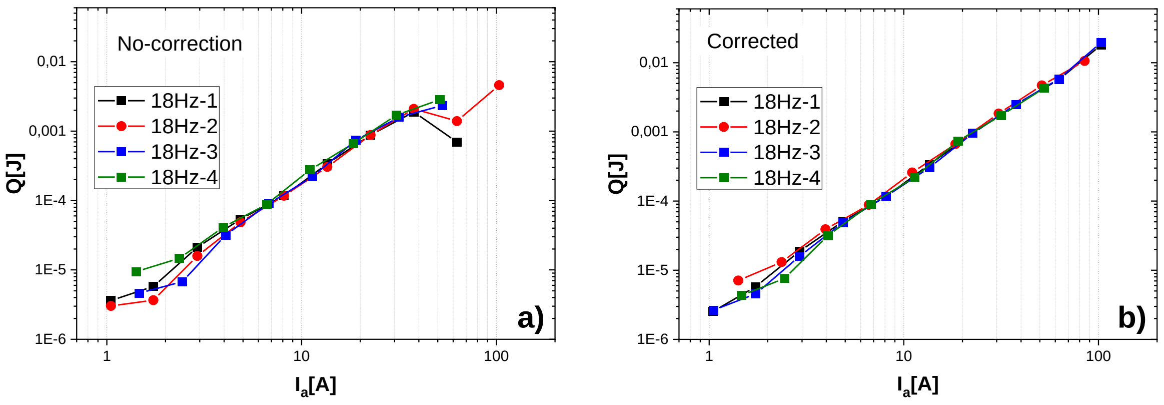

Figure 2: Additional phase correction is necessary for accurate AC loss measurements in coils. (a) and (b) above are for measurements without and with correction, respectively. Different curves are for sub-sequent measurements under the same conditions.

References

[1] M. Meade, "Advances in lock-in amplifiers," *J. Phys. E: Sci. Instr.*, vol. 15, no. 4, p. 395, 1982.

[2] F. Gömöry, "Characterization of high-temperature superconductors by AC susceptibility measurements," *Supercond. Sci. Technol.*, vol. 10, pp. 523–542, 1997.
